# Experimental Studies Indicate That ST-2223, the Antagonist of Histamine H3 and Dopamine D2/D3 Receptors, Restores Social Deficits and Neurotransmission Dysregulation in Mouse Model of Autism

**DOI:** 10.3390/ph15080929

**Published:** 2022-07-27

**Authors:** Nermin Eissa, Karthikkumar Venkatachalam, Petrilla Jayaprakash, Priya Yuvaraju, Markus Falkenstein, Holger Stark, Bassem Sadek

**Affiliations:** 1 Zayed Center for Health Sciences, United Arab Emirates University, Al Ain P.O. Box 17666, United Arab Emirates; nermin.eissa@adu.ac.ae (N.E.); karthikkumar@uaeu.ac.ae (K.V.); petrilla.jp@uaeu.ac.ae (P.J.); priyay@uaeu.ac.ae (P.Y.); 2 Department of Biomedical Sciences, College of Health Sciences, Abu Dhabi University, Abu Dhabi P.O. Box 59911, United Arab Emirates; 3 Department of Pharmacology & Therapeutics, College of Medicine and Health Sciences, United Arab Emirates University, Al Ain P.O. Box 17666, United Arab Emirates; 4 Institute of Pharmaceutical and Medicinal Chemistry, Heinrich Heine University Düsseldorf, Universitaetsstr. 1, 40225 Düsseldorf, Germany; mafal104@hhu.de (M.F.); stark@hhu.de (H.S.)

**Keywords:** social deficits, neurotransmitters, histamine H3R antagonist, dopamine D2R/D3R antagonist, aripiprazole, autism

## Abstract

Altered regulation of neurotransmitters may lead to many pathophysiological changes in brain disorders including autism spectrum disorder (ASD). Given the fact that there are no FDA-approved effective treatments for the social deficits in ASD, the present study determined the effects of chronic systemic treatment of the novel multiple-active H_3_R/D_2_R/D_3_R receptor antagonist ST-2223 on ASD-related social deficits in a male Black and Tan Brachyury (BTBR) mice. ST-2223 (2.5, 5, and 10 mg/kg, i.p.) significantly and dose-dependently mitigated social deficits and disturbed anxiety levels of BTBR mice (*p* < 0.05) in comparison to the effects of aripiprazole (1 mg/kg, i.p.). Moreover, levels of monoaminergic neurotransmitters quantified by LC-MS/MS in four brain regions including the prefrontal cortex, cerebellum, striatum, and hippocampus unveiled significant elevation of histamine (HA) in the cerebellum and striatum; dopamine (DA) in the prefrontal cortex and striatum; as well as acetylcholine (ACh) in the prefrontal cortex, striatum, and hippocampus following ST-2223 (5 mg/kg) administration (all *p* < 0.05). These in vivo findings demonstrate the mitigating effects of a multiple-active H_3_R/D_2_R/D_3_R antagonist on social deficits of assessed BTBR mice, signifying its pharmacological potential to rescue core ASD-related behaviors and altered monoaminergic neurotransmitters. Further studies on neurochemical alterations in ASD are crucial to elucidate the early neurodevelopmental variations behind the core symptoms and heterogeneity of ASD, leading to new approaches for the future therapeutic management of ASD.

## 1. Introduction

The normal development of the brain memory, behavior regulation, and motor activity involve the crucial role of neurotransmitters that interconnect neurons [1]. Evidence suggests that the dysfunction of the neurotransmitter system by affecting neuronal cell migration, differentiation and synaptogenesis, and eventually developmental processes of the brain are thought to be the cause or precocious biomarkers of autism spectrum disorder (ASD) [2,3]. The neuropsychiatric developmental disorder ASD is characterized by social avoidance and lack of interest in social interactions. Deficits in these social domains can affect a child’s ability to function typically, which negatively affects the overall quality of life. Understanding the underlying mechanisms contributing to social deficits in ASD may help inform evidenced-based intervention strategies that may improve the social acceptability of ASD [4], and other dysregulated social interaction-associated disorders. Growing evidence suggests that in addition to genetic and environmental factors, several neurotransmitters are significantly involved in early stages of brain development. Acetylcholine (ACh), serotonin (5-HT), dopamine (DA), γ-aminobutyric acid (GABA), glutamate (Glu), and histamine (HA) are reported to be implicated in the onset and progression of ASD [5]. It is well known that ACh, DA, and HA are neurotransmitters that play a critical role in cognitive functions [6,7]. Altered nicotinic ACh receptor (nAChR) subtypes in brain of autistic individuals are suggested by neuropathologic studies. Loss of nicotinic receptors have been reported in chemical and histochemical studies in the brains of individuals with ASD [8]. Postmortem studies demonstrated a significant decrease in α-7 receptor mRNA levels in frontal cortex of ASD individuals [9]. Moreover, parietal neocortex investigations showed reduced number of neuronal α-4 and β-2 nicotinic acetylcholine receptor (nAChR) subunit. Another study disclosed reduction in the expression of α-4 nAChR subunit in the frontal cortex and increased expression in the cerebellum [10]. In addition, strategies that increase ACh appear to improve cognitive deficits in multiple neuropsychiatric disorders and ASD. Rivastigmine, donepezil, and galantamine (anticholinesterase inhibitors) have shown improvement in cognitive function in patients with Alzheimer’s disease (AD) by increasing synaptic ACh and consequently activating nicotinic and muscarinic receptors [11]. Moreover, selective α4β2 and α7 nAChR subtype agonists AZD3480 and EVP-6124, respectively, demonstrated cognitive performance and attention improvement in patients with attention deficit hyperactivity disorder (ADHD) [12] and schizophrenia (SCH) [13]. Concerning ASD, preclinical studies have shown that the BTBR mouse, a model shown to display core ASD phenotypes, has decreased ACh in the medial prefrontal cortex [14]. Furthermore, BTBR mice showed improvement in sociability and social interactions after administration of acetylcholinesterase inhibitors but did not alter repetitive behavior [15]. It has been reported that BTBR mice compared to normo-social C57 (B6) mice show reduced levels of ACh in the medial prefrontal cortex [14], indicating that the cholinergic transmission deficits may be associated with the abnormal social behaviors witnessed in this strain. Accumulating evidence implicates the cholinergic system, receptors, and neurotransmitters in pathophysiology of ASD; however, extensive research is needed.

In our study, several neurotransmitters have been investigated, and their dysregulation has been shown to be involved in the behavioral phenotype of ASD in BTBR mice. Disruptions to the DA system have been implicated in several neuropsychiatric disorders, including ADHD and recently, ASD [16]. Recent studies have reported reduced dopaminergic mesolimbic circuit signaling in ASD patients highlighting reward-processing deficits, which occurs for both social and nonsocial rewards [17,18]. Moreover, reduced release of DA in the prefrontal cortex was observed in autistic individuals. Significant reductions in both pre- and postsynaptic DA D_2_ and adenosine A_2_A receptor functions were observed in BTBR mice that recapitulate ASD-like phenotypes. Interestingly, recognizing the wake-promoting capacity of histamine H_3_R antagonists in combination with the “caffeine-like effects” of adenosine A_1_R/A_2_AR antagonists, multitargeting ligands were developed to achieve comprehensive therapies of motor disorders such as Parkinson’s disease (PD). Such developed multiple-active compounds were found to simultaneously target A_1_R/A_2A_R/H_3_R with nanomolar affinities profile and could improvel-dopa-induced dyskinesia in rats [19]. Moreover, multiple-targeting approaches for multifaceted neuropsychiatric disorders were the focus of our and other research groups by considering the polypharmacology of targeted receptors while designing the multiple-active compounds [19,20,21,22,23].

DA is the neurotransmitter predominately associated with reward processing [24]. It was observed that impairment in striatal DA neurotransmission correspond with some of the behavioral phenotypes observed in ASD [16]. Up to date, dopamine receptors (DRs) antagonists are the only drugs with consistent clinical efficacy in in ASD. Among these are risperidone and aripiprazole (APZ) that are approved by the Food and Drug Administration (FDA) for the management of ASD-associated behavior, mainly for treating irritability; however, several studies assessed their effects on the core symptoms of ASD. Although not seen in all studies [25], risperidone demonstrated a significant effect on stereotyped behavior in autistic individuals [26,27], and improved social behavior, as evaluated by the Childhood Autism Rating Scale [25,28]. Since the autistic-like behavior arises from dopaminergic dysfunction, the effect of dopamine antagonists observed on the autistic core traits supports that DA modulators should lead to both social and nonsocial behavioral improvement [29]. As demonstrated by previous data, the symptoms of ASD may be due to or exacerbated by abnormal DA signaling [30,31], hence the study of dopaminergic dysfunction is vital as it relates to this neurodevelopmental disorder. In addition, histamine (HA) has been reported to have behavioral effects in brain disorders including Alzheimer’s disease (AD), SCH, narcolepsy, Tourette’s syndrome (TS), and anxiety, all of which overlap with ASD [16,32,33]. Preliminary clinical and preclinical studies suggest that histamine receptors (HRs) 1–3 antagonism reduces symptoms and specific behaviors in ASD patients and relevant animal models [34]. Histamine synthesis occurs predominantly in the tuberomammillary nucleus (TMN), where histidine decarboxylase (*HDC*) is largely expressed. Hence, the premature termination codon (W317X) in HDC gene detected in TS implicates the histaminergic system (HS) in the outcome of this syndrome identifying a significant relationship between TS and ASD reported by several population-based studies [4]. This suggested dysregulation of HS in ASD. Along these lines, we will further explore the effect of dysregulation of ACh, DA, and HA on social behavioral deficits in a BTBR mice model of ASD. Therefore, in this study, we investigated the effects of a recently developed multi-active ligand, namely ST-2223 [*N*-(4-(4-(2-methoxyphenyl) piperazin-1-yl)butyl)-6-(3-(piperidin-1-yl)propoxy)-2-naphthamide], having histamine H_3_ receptor (H3R) antagonist affinity (*h*H_3_R *K*_i_ = 4.6 nM) and balanced dopamine D_2_R/D_3_R antagonistic properties, on social deficits in a BTBR mouse model of ASD (Table 1). LC-MS/MS was carried out to quantify the levels of ACh, DA, and HA in mice-specific brain tissues, to determine the effect of chronic treatment of ST-2223 on the levels of these neurotransmitters and the associated behavioral improvements observed in BTBR mice.

## 2. Results

### 2.1. Effects of Systemic Administration of ST-2223 on Sociability Deficits in BTBR Mice

**Figure 1:**
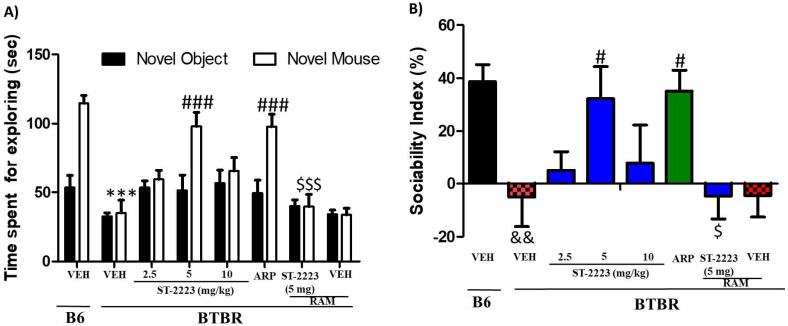
Effects of ST-2223 on the sociability deficits of BTBR mice in sociability approach test. (**A**) B6 and BTBR mice spent time for exploring novel object/novel mouse. (**B**) Sociability index (SI). *** *p* < 0.001 compared to VEH-treated B6 mice. ^#^ *p* < 0.05, ^###^ *p* < 0.001 compared to VEH-treated BTBR mice. ^$^ *p* < 0.05, ^$$$^ *p* < 0.001 compared to ST-2223-(5 mg)- pretreated BTBR mice. ^&&^ *p* < 0.01 compared to VEH-treated B6 mice. Data are expressed as the mean ± SEM (n = 7).

The chronic systemic administration of ST-2223 at three different doses (2.5, 5, and 10 mg/kg, i.p.) and aripiprazole (ARP) (1 mg/kg, i.p.) on ASD-like deficits of sociability approach in social approach test (SAT) in BTBR mice are shown in Figure 1 The results of the two-way ANOVA showed that there was a significant main effect for strain, treatment, and also for strain × treatment interaction on observed SI values, with (*F*_(1,84)_ = 39.52, *p* < 0.01), (*F*_(6,84)_ = 6.82, *p* < 0.001), and (*F*_(6,84)_ = 2.28, *p* < 0.05), respectively. Statistical analysis results revealed that chronic systemic treatment with ST-2223 and ARP prior to SAT has markedly increased sociability, measured by more time expended exploring the novel mouse (NM) compared to the time expended exploring the novel object (NO), with [*F*_(7,48)_ = 16.81; *p* < 0.001] (Figure 1A). As observed in the post hoc analyses, BTBR mice spent the same amount of time with NO and NM [*F*_(1,12)_ = 0.07; *p* = 0.81], when compared to vehicle (VEH)- treated B6 control mice, spending significantly more time with NM over NO, with [*F*_(1,12)_ = 34.76; *p* < 0.001] (Figure 1A). ST-2223 (5 mg/kg) significantly enhanced time spent with the NM by BTBR mice, with [*F*_(1,12)_ = 11.26; *p* < 0.01], which was similar to that exerted by ARP (1 mg/kg), with [*F*_(1,12)_ = 0.14; *p* = 0.71] (Figure 1A). However, chronic systemic treatment of BTBR mice with ST-2223 (2.5 and 10 mg/kg) was unsuccessful to exert remarked improvement in the time spent with the NM vs. NO, with [*F*_(1,12)_ = 0.529; *p* = 0.48] and [*F*_(1,12)_ = 0.41; *p* = 0.53], respectively (Figure 1A). Furthermore, neither B6 control vs. ST-2223 (5 mg/kg) nor B6 control vs. ARP (1 mg) displayed significant differences (*p* = 0.68 and *p* = 0.27, respectively, concerning the time spent with NM) (Figure 1A).

As seen in Figure 1A, and when comparing the exploring time for NM between the groups, and following post hoc analyses, BTBR mice spent less time with NM, [*F*_(1,12)_ = 53.54; *p* < 0.001], as compared to VEH-exposed control B6 animals (Figure 1A). ST-2223, 2.5 mg/kg evidenced improvements in sociability expressed as an increased exploratory time spent with NM, and when comparing to VEH-treated BTBR mice, however, it was not a significant level [*F*_(1,12)_ = 4.54; *p* > 0.05] (Figure 1A). ST-2223 (5 mg/kg [*F*_(1,12)_ = 30.15; *p* < 0.001,) and ARP (1mg/kg, i.p.) [*F*_(1,12)_ = 25.54; *p* < 0.001] significantly improved exploring time spent with NM when compared with VEH-treated BTBR mice (Figure 1A. As observed in the post hoc analyses by Tukey test, the ST-2223 provided improvement of exploring time spent with NM was reversed following co-administration with the CNS-penetrant H_3_R agonist (R-alpha methyl histamine) RAM (10 mg/kg, i.p.), with [*F*_(1,12)_ = 28.16; *p* < 0.001] (Figure 1A). Moreover, VEH-treated BTBR and BTBR mice pretreated with 2.5 and 10 mg/kg of ST-2223 exhibited similar adjustment to change in the sociability index (SI) (all *p* values > 0.05), indicating that the significant improvement in sociability exhibited by BTBR mice was restricted to ST-2223 (5 mg/kg) treatment t (*p* < 0.05). (Figure 1B). Additionally, the observed results for SI values showed that the ST-2223—(5 mg/kg)-provided enhancement on sociability performance was completely abrogated when co-administered with RAM (10 mg/kg, i.p.), with (*p* < 0.05) for the comparison of groups treated with ST-2223-(5 mg/kg) and ST-2223 (5 mg) + RAM (Figure 1B).

### 2.2. Effects of Systemic Administrtion of ST-2223 on Social Preference in BTBR Mice

**Figure 2:**
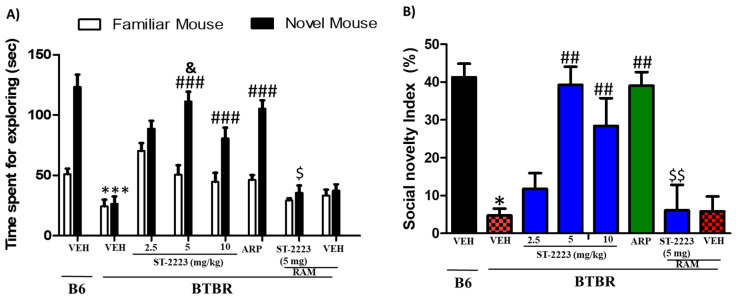
Effects of ST-2223 on social novelty deficit of BTBR mice in social novelty approach test (**A**) B6 and BTBR mice spent time for exploring novel mouse/familiar mouse. (**B**) Social novelty index (SNI). * *p* < 0.05, *** *p* < 0.001 compared to VEH-treated B6 mice. ^##^ *p* < 0.01, ^###^ *p* < 0.001 compared to VEH-treated BTBR mice. ^&^ *p* < 0.05 compared to ST-2223 at doses of 2.5 and 10 mg/kg. ^$^ *p* < 0.05, ^$$^ *p* < 0.01 compared to ST2223-(5 mg)-treated BTBR mice. Data are expressed as the mean ± SEM (n = 7).

The effects of intraperitoneal chronic administration of three different doses of ST-2223 (2.5, 5, and 10 mg/kg) and ARP (1 mg/kg) on the time spent with the familiar mouse (FM) and the novel mouse (NM) are shown in Figure 2. The results of post hoc analyses indicated that control B6 mice showed significant interactions with NM vs. FM, with [*F*_(1,12)_ = 42.36; *p* < 0.001] (Figure 2A). As seen in Figure 2A and following post hoc analyses, BTBR mice spent less time for exploring NM, with [*F*_(1,12)_ = 67.42; *p* < 0.001], as compared to B6 control mice. However, BTBR mice pretreated with ST-2223 (5 and 10 mg/kg, i.p.), ARP (1 mg/kg) showed enhanced preference to stranger NM over FM [*F*_(1,12)_ = 72.02; *p* < 0.001], [*F*_(1,12)_ = 25.67; *p* < 0.001], [*F*_(1,12)_ = 72.36; *p* < 0.001], respectively (Figure 2A) and when compared to VEH-treated BTBR mice. Moreover, ST-2223 (5 mg/kg) provided significantly higher improvement of social novelty preference than that witnessed with ST-2223 (2.5 mg/kg), with [*F*_(1,12)_ = 57.36; *p* < 0.001]. Furthermore, no significant difference was observed between control B6 and ST-2223 (5 mg) treated mice in exploratory time for NM (*p* = 0.37) (Figure 2A). Similar to the results observed on the ST-2223—provided effects on sociability, the ST-2223-provided effect on social novelty preference was completely reversed by co-administration of H_3_R agonist RAM [*F*_(1,12)_ = 57.93; *p* < 0.001] (Figure 2A).

The results observed for SNI showed that BTBR mice exhibited significantly lower social novelty preference index as compared to B6 control mice (*p* < 0.01). No significant difference was found in the social novelty index (SNI) between control B6 and ST-2223 (5 mg/kg, i.p.) treated BTBR mice, indicating that BTBR mice exhibited significantly improved social novelty performance when pretreated with ST-2223 (5 mg/kg) (Figure 2B). Additionally, the results for SNI values showed that the ST-2223—(5 mg)-provided enhancement in social novelty performance was completely counteracted when the centrally active H_3_R agonist RAM (10 mg/kg, i.p.) was co-administered [*F*_(1,12)_ = 15.951; *p* < 0.01] for the comparison of groups treated with ST-2223—(5 mg/kg) and ST-2223 (5 mg) + RAM (Figure 2B). Similar to the results observed for SI values, statistical analyses of the two-way ANOVA showed that there was a significant main effect for strain, treatment, and also for strain × treatment interaction on observed SNI values, with (*F*_(1,84)_ = 89.06, *p* < 0.001), (*F*_(6,84)_ = 6.82, *p* < 0.001), and (*F*_(6,84)_ = 3.82, *p* < 0.01), respectively.

### 2.3. Effects of Systemic Administration of ST-2223 on Anxiety and Locomotor Activity in BTBR Mice in OFL Test

**Figure 3:**
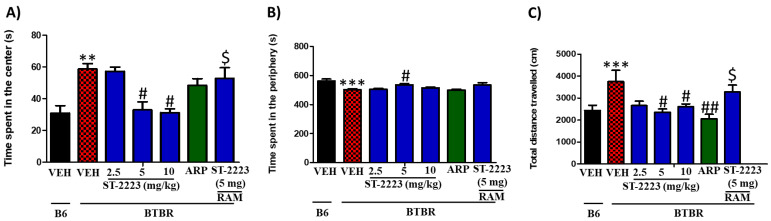
Effects of ST-2223 on anxiety-like behaviors and locomotor activity of B6 and BTBR mice in open field test. Time spent in the Center (**A**). Time spent in the periphery (**B**). Total distance travelled (**C**). ** *p* < 0.01 compared to VEH-treated B6 mice. *** *p* < 0.001 compared to VEH-treated B6 mice. ^#^ *p* < 0.05 compared to VEH-treated BTBR mice. ^##^ *p* < 0.01 compared to VEH-treated BTBR mice. ^$^ *p* < 0.05 compared to ST-2223-(5 mg)- treated BTBR mice. Data are expressed as the mean ± SEM (n = 5).

OFL test was employed to evaluate the effects of the applied three doses of ST-2223 on the anxiety-like behaviors and locomotion of treated mice (Figure 3A–C). As shown in the results observed in Figure 3A, BTBR mice exhibited significantly more time in the center of the arena [*F*_(1,8)_ = 16.55, *p* < 0.01] when compared with control B6 mice. Analyses of variances revealed that the BTBR mice when treated with ST-2223 (5 and 10 mg/kg, i.p.) expended significantly less time in the central compartment of the open field, with [*F*_(1,8)_ = 10.85, *p* < 0.05] and [*F*_(1,12)_ = 7.39, *p* < 0.05], respectively (Figure 3A). However, systemic chronic treatment with the lower (2.5 mg/kg) dose of ST-2223 failed to decrease the time spent in the center of the arena to a significant level, with [*F*_(1,8)_ = 0.02, *p* = 0.84] (Figure 3A). As shown in the results observed for the time spent in the periphery, BTBR mice exhibited a significant decrease in the time spent in the periphery, with [*F*_(1,8)_ = 16.96, *p* < 0.001] (Figure 3B). However, and like the results observed for the time spent in the center of the arena, treatment with ST-2223 (5 mg/kg, i.p.) significantly corrected the decreased time [*F*_(1,8)_ = 10.65, *p* < 0.05), and as compared with BTBR mice. Notably, after ST-2223 (2.5 and 10 mg/kg, i.p.) as well as ARP (1 mg/kg, i.p.) treatments, there were no group differences in modulating the time spent by BTBR mice in the periphery, with [*F*_(1,8)_ = 1.82, *p* = 0.21] and [*F*_(1,8)_ = 0.05, *p* = 0.83], respectively (Figure 3B). Interestingly, chronic systemic co-administration of BTBR with RAM (10 mg/kg, i.p.) reversed the ST-222(5 mg)—provided effects on time spent in the center, with [*F*_(1,8)_ = 0.65, *p* = 0.44]], for the comparison of ST-2223(5 mg) + RAM(10 mg)- treated group with saline-treated BTBR control mice (Figure 3A). Additionally, the results observed showed that the total distance travelled by BTBR mice was significantly higher when compared to control B6 mice, with [*F*_(1,8)_= 32.96, *p* < 0.001]. However, ST-2223 (5 and 10 mg/kg) and ARP (1 mg/kg) significantly decreased the total distance travelled, with [*F*_(1,8)_= 6.79, *p* < 0.05], [*F*_(1,8)_= 5.48, *p* < 0.05], and [*F*_(1,8)_= 9.33, *p* < 0.01], respectively, and as compared to VEH-treated BTBR mice (Figure 3C).

### 2.4. Effects of ST-2223 on the Brain Levels of Histamine, Dopamine, and Acetylcholine in Different Brain Parts of BTBR Mice

**Figure 4:**
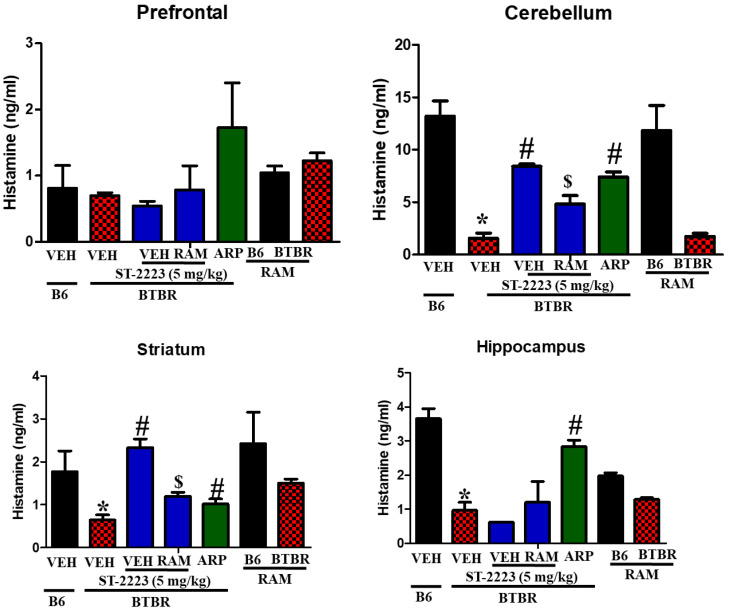
Estimation of histamine levels by LC-MS/MS in different brain regions of BTBR. * *p* < 0.05 compared to VEH- treated B6 mice. ^#^ *p* < 0.05 compared to VEH-treated BTBR mice. ^$^ *p* < 0.05 compared to ST-2223-(5 mg)-treated BTBR mice. Data are expressed as the mean ± SEM (n = 5).

The BTBR mice demonstrated low levels of histamine in the assessed brain regions when compared to the control B6 animals, especially in the cerebellum, striatum, and hippocampus tissues, with [*F*_(1,8)_ = 57.65; *p* < 0.05], [*F*_(1,8)_ = 6.37; *p* < 0.05], and [*F*_(1,8)_ = 44.84; *p* < 0.05], respectively (Figure 4). However, there was no significant change in the levels of histamine assessed in the prefrontal cortex with [*F*_(1,8)_ = 0.10; *p* = 0.77]. Chronic systemic treatment of BTBR mice with ST-2223 (5 mg/kg, i.p.) significantly increased the levels of histamine in cerebellum and striatum [*F*_(1,8)_ = 131.21; *p* < 0.05], [*F*_(1,8)_ = 45.10; *p* < 0.05], without significant enhancement of brain histamine in hippocampal tissues with [*F*_(1,8)_ = 2.77; *p* = 0.14]. Interestingly, the reference drug ARP (1 mg/kg, i.p.) increased the levels of histamine in the cerebellum, striatum, and hippocampus, with [*F*_(1,8)_ = 68.38; *p* < 0.05], [*F*_(1,8)_ = 6.43; *p* < 0.05] and [*F*_(1,8)_ = 37.51; *p* < 0.05], respectively, which was comparatively higher than VEH-treated BTBR mice. Notably, co-administration of RAM (10 mg/kg, i.p.) and ST-2223, reversed the ST-2223-provided increment of histamine levels in cerebellum and striatum with ([*F*_(1,8)_ = 18.00; *p* < 0.05] and [*F*_(1,8)_ = 25.42; *p* < 0.05], respectively.

**Figure 5:**
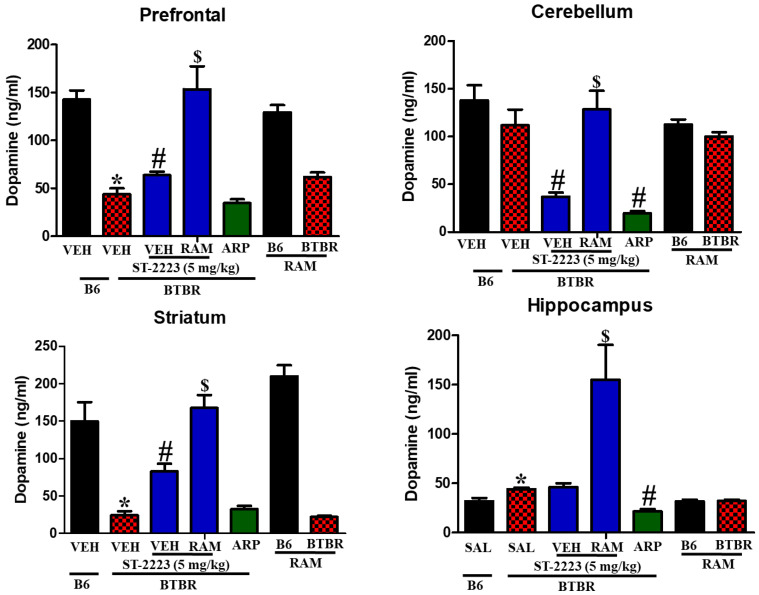
Estimation of dopamine levels by LC-MS/MS in different brain regions of BTBR * *p* < 0.05 compared to VEH-treated B6 mice. ^#^ *p* < 0.05 compared to VEH-treated BTBR mice. ^$^ *p* < 0.05 compared to ST-2223-(5 mg)- treated BTBR mice. Data are expressed as the mean ± SEM (n = 5).

Moreover, the levels of dopamine were evaluated in the same regions of treated BTBR mice (Figure 5). The observed results showed that dopamine levels in the control mice were significantly higher than that assessed in brain regions of BTBR mice, and this is specifically in prefrontal cortex [*F*_(1,8)_ = 69.41; *p* < 0.05] and striatum [*F*_(1,8)_ =27.41; *p* < 0.05] (Figure 5). However, administration of ST-2223 (5 mg/kg) significantly increased the dopamine levels in both regions of tested BTBR mice, with ([*F*_(1,8)_ = 8.21; *p* < 0.05] and [*F*_(1,8)_ = 26.17; *p* < 0.05], respectively. Unexpectedly, chronic systemic administration with H_3_R agonist RAM failed to reverse the ST-2223-provided increase on dopamine levels, but rather significantly further increased the enhanced levels measured for dopamine in ST-2223-pretreated BTBR mice, with [*F*_(1,8)_ = 17.24; *p* < 0.05] and [*F*_(1,8)_ = 19.40; *p* < 0.05], respectively.

**Figure 6:**
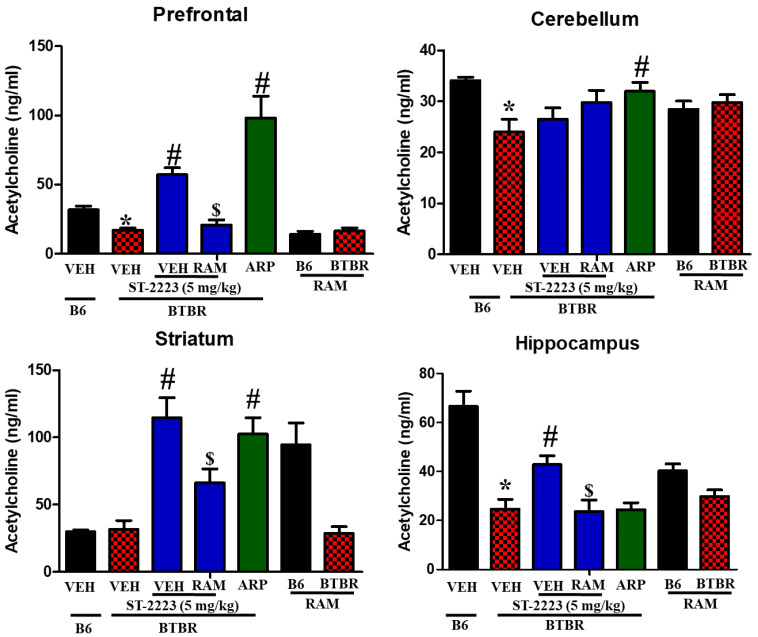
Estimation of acetylcholine levels by LC-MS/MS in different brain regions of BTBR. * *p* < 0.05 compared to VEH-treated B6 mice. ^#^ *p* < 0.05 compared to VEH-treated BTBR mice. ^$^ *p* < 0.05 compared to ST-2223-(5 mg)- treated BTBR mice. Data are expressed as the mean ± SEM (n = 5).

The levels of acetylcholine were shown in the four assessed brain regions of BTBR mice (Figure 6). The results observed clearly that acetylcholine levels were significantly lower in BTBR mice compared to control B6 mice, particularly in prefrontal cortex, cerebellum, and hippocampus tissues, with [*F*_(1,8)_ = 23.89; *p* < 0.05], [*F*_(1,8)_ = 11.57; *p* < 0.05], and [*F*_(1,8)_ = 32.45; *p* < 0.05], respectively (Figure 6). However, and following chronic systemic administration of BTBR mice with ST-2223 (5 mg/kg), a significant increase of acetylcholine levels in prefrontal cortex [*F*_(1,8)_ = 49.99; *p* < 0.05], striatum [*F*_(1,8)_ = 34.55; *p* < 0.05], and hippocampus [*F*_(1,8)_ = 11.24; *p* < 0.05] were witnessed. Similarly, the reference drug ARP (1 mg/kg) demonstrated a signified increase in the levels of acetylcholine in the prefrontal cortex, cerebellum, and striatum tissues, with [*F*_(1,8)_ = 26.34; *p* < 0.05], [*F*_(1,8)_ = 7.45; *p* < 0.05], and [*F*_(1,8)_ = 22.89; *p* < 0.05], respectively, and these enhancements were entirely nullified following systemic chronic co-administration with the CNS-penetrant H_3_R agonist RAM (10 mg/kg), with [*F*_(1,8)_ = 27.74; *p* < 0.05], [*F*_(1,8)_= 7.55; *p* < 0.05], and [*F*_(1,8)_ = 10.18; *p* < 0.05], respectively, and as compared with brain regions of ST-2223 (5 mg)- treated BTBR mice (Figure 6). Table 2 summarizes the overall effects observed for the test compound ST-2223 and the reference drug ARP on histamine, dopamine, and acetylcholine in brain regions of the assessed mice (Table 2).

## 3. Discussion

Brain histaminergic, dopaminergic, and cholinergic neurotransmission alterations are suggested to play a crucial role in ASD-related behavioral features as reported in clinical studies [16,21,38]. Consequently, the aim of the current study was to assess the modulating effects of the novel multiple-active test compound ST-2223 on brain HA, DA, and ACh on ASD-behavioral symptoms displayed by BTBR mice model of ASD. In vitro, ST-2223 was evaluated for its H_3_R affinity on membrane preparations of HEK-293 cells, which stably express the hH_3_R, by [^3^H]*N*^α^-methylhistamine displacement assays. The results demonstrated that ST-2223 had high in vitro antagonist affinity for the desired targets, *h*H_3_Rs (*K*_i_ = 4.6 nM), *h*D_2_Rs (*K*_i_ = 19.8 nM), and hD_3_Rs (*K*_i_ = 2.0 nM), with a ratio of *h*D_2_Rs/*h*D_3_Rs of 10. Additionally, the ST-2223 observed in vitro results exhibited low affinity for *h*H_1_Rs (*K*_i_ = 85.2 nM), hD_1_Rs (*K*_i_ = 564 nM), *h*D_5_Rs (*K*_i_ = 5064 nM), and neglectful inhibition of acetylcholine esterase enzyme (eeAChE) (Table 1). Effective pharmacological treatments for social deficits in ASD are required. Our findings shows that ST-2223 administration improves social interaction deficits significantly and dose-dependently in BTBR mice with naturally occurring lower sociability. In SAT, chronic systemic pretreatment with ST-2223 modulated the impairment in sociability and social novelty paradigms demonstrated by BTBR, with significant SI and SNI, comparable to the control B6 mice. Several previous studies have reported the procognitive effects of several H_3_R antagonists on social memory [39,40,41,42], an altered behavioral characteristic observed in ASD [41]. Our observations revealed that the sociability as well as social preference behavior-enhancing effects of ST-2223 were dose-dependent, since ST-2223 (5 mg/kg) exhibited an optimal effect that is comparable to that provided by the reference drug APZ, which has recently been shown to exhibit a slow monophasic dissociation at the D_2_Rs and D_3_Rs [43]. However, a dose of 10 mg/kg failed to significantly improve upon the ST-2223 (5 mg)- provided sociability and social novelty enhancement. The observed dose-dependent effects of systemic administration of ST-2223 are consistent with our previous studies with non-imidazole-based H3R antagonists on VPA-induced ASD in Tuck-Ordinary and B6 mice [44,45]. Moreover, similar sociability-enhancing effects for ST-2223 was observed in an experimental study with the imidazole based H3R antagonist ciproxifan in Swiss mice [38,46]. In further abrogative studies, the optimal ST-2223 provided improvement in sociability and social novelty were entirely counteracted when mice were co-administered with the brain-penetrant H3R agonist RAM, implicating the important role of HA and histaminergic system in modulating alteration of sociability processes in BTBR mice in SAT. The results of the study supported our hypothesis that the potential effect of ST-2223 on social parameters is due to multiple neurotransmitter release other than HA, such as DA and ACh, in specific brain regions, which might be based on the inhibition of the H3R hetero-receptors [47]. This is in addition to its simultaneous D2R/D3R antagonist properties that result in modulation of abnormal dopaminergic transmission. ST-2223 enhanced the DA level in the striatum and prefrontal cortex, which was not reversed by RAM. Further studies are necessary to examine the effect of an enhanced level of DA in all brain regions by co-administration of ST-2223 (5 mg) and RAM. The optimal dose of ST-2223(5 mg/kg) has exerted its effects on the dopaminergic system through elevating DA levels in the striatum and prefrontal, while inhibiting the level in cerebellum of BTBR mice. This finding may suggest that the effect of ST-2223 on dopaminergic activity may be based on D_2_R partial agonism when dopamine release is low, and it would be expected to suppress dopaminergic activity when dopamine release at D_2_R is augmented, using a stabilizing mechanism of action such as that of ARP on the dopaminergic system [48,49]. The observed enhanced dopamine release in prefrontal cortex has mirrored a previous study with both BF2.649 [50] and GSK189254 [51] in rat prefrontal cortex. Taken together, these studies support the therapeutic potential of H_3_R antagonists to treat negative symptoms and cognitive deficits associated withSCH, as defined by hypodopaminergic function in prefrontal cortex. Additionally, these findings highlight the interplay of DA and HA in cognitive deficits that may include social problems observed in ASD. These data suggest that ST-2223 may have a potential therapeutic role in the management of social deficiencies in ASD and other brain disorders associated with cognitive deficits. Interestingly, a recent study that employed phenotypic BTBR mice demonstrated alleviated social approaching, non-selective and object-based attention, upon intranasal administration of DA, likely by enhancing the level of tyrosine hydroxylase in the striatum that consequently elevate the concentration of DA in this brain region [52]. This reported observation supports our current finding, suggesting that treatment with DA may present a promising therapy for diverse types of ASD. On the other hand, the ARP failed to affect extracellular levels of DA in all brain regions, in contrary to a previous finding reporting that ARP produced a significant increase of DA levels in dialysate after the administration of a 0.3 mg/kg of ARP in the prefrontal cortex of B6 mice [53]. This reveals that ARP can, in fact, elevate the cortical DA, thus challenging previously reported failures (at high doses) of this drug to affect DA activity in cortical regions [54], including our current result. Despite the evidence for DA deficiency in various brain regions in BTBR and the failure of ARP to restore it, the social parameters’ improvement in BTBR mice, observed by ARP administration may be due to its enhancement of HA in cerebellum and hippocampus, or ACh in cerebellum and striatum. Previous reports suggested that an impaired cholinergic system causes cognitive deficits that may include socialimpairements, which were reversed by donepezil treatments [55,56]. Moreover, major finding of a previous study demonstrated the association between enhancement of ACh levels and significant improvement in cognitive rigidity and social deficiencies in BTBR mice expressing low brain ACh levels [15]. Therefore, considering the levels of different brain neurotransmitters, including HA, DA, and ACh, in various brain areas was necessary to understand the role of neurotransmitters involved in the observed social behavior of the BTBR mice with ASD-like behaviors, as well as post-treatment with ST-2223. In support of this view, considering the role of DA in ASD, a recent human neuroimaging research has consistently revealed analogously reduced functional responses in the reward circuits in ASD individuals while processing rewards, including social rewards [18,57,58]. Moreover, another study provided indirect support to our current results through displaying the ability of drugs targeting D2Rs as ARP to modulate the core symptoms of ASD [18,59,60], in addition of key contribution of D2Rs in mediating social behavior in humans [61,62]. Collectively, the numerous in vivo evidence that has indicated the unique potential cognition-enhancing property of H3R antagonists that aligns with our results [5,21,32,44,63,64,65], demonstrated through mediating social deficiencies, suggests the potential role of the multiple-acting compound ST-2223 that may open a new venue for therapeutic interventions in ASD individuals.

In agreement with the role of H3R/D2R/D3R antagonist on the effects of core autism-like behaviors, we found here that treatment of mice with ST-2223 (5 mg/kg) was accompanied by modulating abnormal anxiety, as well as restoring hyperactivity exhibited by BTBR mice. These results comprehend our previously observations for ST-2223 in open-field assessment [46]. Moreover, the abnormal anxiety level observed by BTBR mice by spending more time in the center of the chamber reflects attention deficit and impulsive behavior. This observation may be related to decline level of ACh in prefrontal cortex, as stated by a previous study that highlighted the importance of ACh in attention and cognition [14]. This interpretation is in line with the recorded decline of ACh in the prefrontal cortex of tested BTBR mice. The ST-2223 enhancement of ACh in the prefrontal cortex suggests the mediation of other neurotransmitters than HA, such as ACh, through H_3_R heteroreceptors. Additionally, the ST-2223 augmentation of ACh in cerebellum and HA in hippocampus documented by our data are implicated in cognitive enhancement and consequent social improvement and are consistent with diverse preceding research that centered on the procognitive effects of several H3R antagonists on social reminiscence [39,40,41,42], involving H_3_ auto- and hetero-receptors. The effects of systemic management with ST-2223 on locomotion was assessed simultaneously via OFL test to exclude possible intrinsic effects of spontaneous locomotor activity that may give rise to a false-positive results in the social behavioral paradigms observed. Accordingly, the enhancements in sociability and social novelty observed for ST-2223 in SAT appear unlikely to be accompanied with a modulating effect in locomotor activity of the examined mice. Additionally, in accordance with the abrogated ST-2223 (5 mg)- provided effects on social parameters with RAM coadministration, the ST-2223 (5 mg)- provided effects on anxiety-like behaviors was reversed when mice were co-administration with RAM, demonstrating that ST-2223 may have exerted its effects on hyperactivity and anxiety-like behaviors through strongly correlating the regulation of both HA and ACh neurotransmitters with anxiety observed in BTBR mice. These outcomes are in accordance with preceding results that found out anxiolytic-like effects of a non-imidazole-based H3R antagonist, as well as dual active compound E100 in a mice model of ASD [46,66]. On the other hand, ARP failed to restore the abnormal anxiety observed by BTBR mice. This is in contrast with different preclinical studies that demonstrated considerable anxiolytic monotherapy effect of ARP [67,68,69]. This failure of restoration may be due to the association of the impulsive behavior with anxiety in BTBR mice resulting in the abnormal anxiety exhibited. A previous study provides support for this view, as ARP did not modify levels of impulsivity in high- and low-impulsive rats on the 5-CSRT task [70], and this absence of effect was suggested to be due to a concomitant action on pre- and postsynaptic dopamine D_2_Rs/D_3_Rs [71]. The positive effect of ST-2223 on abnormal BTBR anxiety displayed shed light on the crucial role of multitarget novel compounds in exerting potential effects compared to monotherapy. Notably, the proposed advantage of developing multiple-active compound as ST-2223 with combined affinities at specific targets is to avoid putative drug-drug interactions that may occur with administration of combined therapy. In addition, co-application of two different drugs necessitates dose finding since the effective doses might be considerably distinctive from the ones applied in the case of monotherapy due to differences in pharmacokinetics of each compound. The current study is complementary to our previous preliminary findings of acute systemic administration of ST-2223, which was a preclinical study that demonstrated the ameliorative effects of ST-2223 on repetitive and restricted behaviors in BTBR mice, another core feature of ASD, through a battery of behavioral tests [72]. However, further studies are necessary to determine pharmacokinetics/pharmacodynamics for ST-2223 to corroborate the provided ameliorating outcomes on ASD-like features and to exclude possible off-target consequences.

## 4. Materials and Methods

### 4.1. Animals

Adult male inbred strains BTBR *T*_ *Itpr3tf*/J (BTBR) and C57BL/6J (B6) mice (aged 8–10 weeks, weighing 25–35 g) were purchased from Jackson Laboratory (Bar Harbor, ME, USA). The mice were bred in the local central animal facility of the College of Medicine and Health Sciences, United Arab Emirates University [73]. Mice were kept on a 12 h/12-h light cycle (lights on at 6 am) in a humidity- and temperature-controlled room (22–25 °C). Water and a standard rodent chow diet were available to the animals throughout, in their home cages. Experiments were conducted during the light cycle. All experimental research involving animals were performed according to the recommendations of the European Communities Council Directive of 24 November 1986 (86/609/EEC) and with the approval of the Institutional Animal Ethics Committee in the College of Medicine and Health Sciences/United Arab Emirates (Approval No. ERA-2017-5603). All authors affirm that all procedures had been executed in accordance with relevant guidelines and regulations. The effect of ST-2223 was not conducted on B6 mice to reduce the number of control animals used, as in our previous study ST-2223 showed no behavioral alterations in B6 control mice [72].

### 4.2. Drugs

ST-2223 [*N*-(4-(4-(2-methoxyphenyl)piperazin-1-yl)butyl)-6-(3-(piperidin-1-yl)propoxy)-2-naphthamide] was prepared and in vitro, profiled in the Institute of Pharmaceutical and Medicinal Chemistry, Heinrich Heine University Düsseldorf, Germany, according to a previously described procedure (Table 1) [35,36,74]. The H3 receptor agonist R-alpha methyl histamine (RAM) (10 mg/kg, i.p.) and the reference drug aripiprazole (APZ) (1 mg/kg, i.p.) were purchased from Sigma-Aldrich (St. Louis, MO, USA). The drugs were prepared daily using 1% DMSO in 0.9% normal saline (vehicle) for intraperitoneal (i.p.) administration, at a volume of 10 mL/kg adjusted to body weight of mice. For LC-MS/MS, the standards acetylcholine (1008501), histamine (1309009), and dopamine (1225204) were purchased from USP (Twinbrook Pkwy, Rockville, MD, USA), while the internal standard acetylcholine D4 1,1,2,2, d4 (D-2558) was purchased from CDN Isotopes (Pointe-Claire, Quebec, Canada).The internal standards histamine-α,α,β,β-d4 dihydrochloride (762962) and dopamine 1,1,2,2- d4 hydrochloride (73483) were procured from Sigma-Aldrich (St. Louis, MO, USA).

### 4.3. Study Design and Treatments

**Figure 7:**
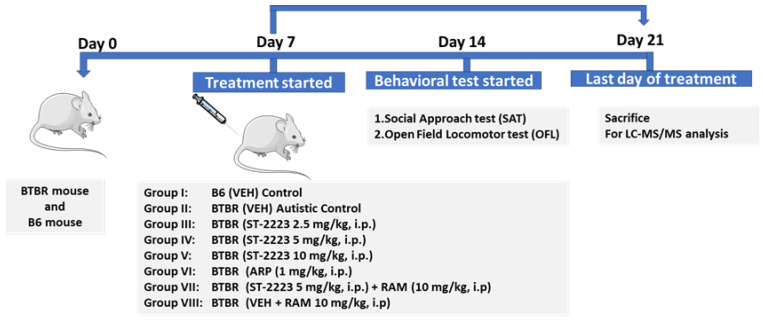
Schematic experimental design.

All the mice were randomly divided into nine groups of 5–7 mice each. The mice were injected intraperitonially once daily for 21 days with different doses of ST2223 (2.5, 5, and 10 mg/kg, i.p.), ARP 1 mg/kg or vehicle (1% DMSO in 0.9% normal saline). Group I, B6 mice were injected with vehicle served as control. Group II, BTBR mice were treated with vehicle. Groups III–V, BTBR mice received i.p. injections of different doses of ST2223 (2.5, 5, and 10 mg/kg, i.p.), respectively. Group VI, BTBR mice were injected with ARP (1 mg/kg, i.p.). For abrogation studies, Group VII, BTBR mice were treated with ST2223 (5 mg/kg, i.p.) along with RAM (10 mg/kg, i.p.). Group VIII, B6 mice were co-injected with vehicle and RAM (10 mg/kg, i.p). All treatments and vehicle (VEH) were administered 30–45 min before each behavioral test. The doses of these drugs were selected based on previous reports [15,75]. Behavioral testing was performed between 9:00 am and 3:00 pm in an order randomized by group and in the following sequence: social approach test (SAT) and open field locomotor test (OFL). The chronic treatment started one week before the behavioral test and was continued for a total of 21 days as described above (Figure 7). Following behavioral tests, i.e., on day 21 of systemic treatment, five of the animals were sacrificed. The animals were deeply anesthetized with pentobarbital (40 mg/kg, i.p., body weight). To wash out the blood, cardiac perfusion was carried out using 0.01 M phosphate-buffered saline (PBS) at pH 7.4. The brains were quickly removed and placed on an ice plate, where the cerebellum, hippocampus, prefrontal cortex, and striatum were excised from the brain and snap-frozen in liquid nitrogen for later LC-MS/MS analysis [17,44].

### 4.4. Behavioral Tests

#### 4.4.1. Social Approach Test (SAT)

The social approach test (SAT) was performed using an automated three-chamber device (EthoVision^®^ Software, Noldus, The Netherlands) as previously described [44,76,77,78]. The transparent polycarbonate chamber is rectangular and is composed of three interconnected partitions (homemade), which are separated by two sliding doors. In brief, after 10 min of habituation, the study mouse was placed in the central chamber with the doors opened, and the mouse was given the choice to interact with either an empty plastic cup located in one side chamber, referred to as a novel object (NO), or a similar plastic cup with an unfamiliar mouse inside it located in the opposite chamber, referred to as a novel mouse (NM), which was matched in age, sex, and strain with test mouse. To avoid innate side preferences, the plastic cup for NO and NM were randomly placed in either chamber and the placement was changed between studies and between mice. For 10 min, the test mouse was allowed to explore all three chambers and cups, and the time spent interacting with NO and NM (sniffing) was automatically recorded by EthoVision^®^ Software. Immediately after this session, a novel mouse is kept in the empty cup and is referred to as novel mouse (NM), and the mouse in the other cup from the earlier session is referred to as familiar mouse (FM). The study mouse is allowed to explore the three chambers for 10 min, and the time spent for interacting with both mice was automatically recorded, to assess social novelty preference. Eight groups of 7 mice/group were used for the SAT. To allow the direct comparison of social behavior between the treated groups, the sociability index (SI) and social novelty index (SNI) were calculated with the following formula, as previously described [44,76]:

$$\mathrm{SI}=\frac{\left(\mathrm{Time}\;\mathrm{exploring}\;\mathrm{NM}-\mathrm{Time}\;\mathrm{exploring}\;\mathrm{NO}\right)}{\left(\mathrm{Time}\;\mathrm{exploring}\;\mathrm{NM}+\mathrm{Time}\;\mathrm{exploring}\;\mathrm{NO}\right)}$$

$$\mathrm{SNI}=\frac{\left(\mathrm{Time}\;\mathrm{exploring}\;\mathrm{NM}-\mathrm{Time}\;\mathrm{exploring}\;\mathrm{FM}\right)}{\left(\mathrm{Time}\;\mathrm{exploring}\;\mathrm{NM}+\mathrm{Time}\;\mathrm{exploring}\;\mathrm{FM}\right)}$$

#### 4.4.2. Open Field Locomotor Test (OFL)

Open field locomotor test (OFL) systematically assesses exploratory activity based on subjecting an animal to an unfamiliar environment whose escape is prevented by surrounding walls. It is a model of anxiety-like behavior, suitable for assessing motor activity as a reaction to an unknown environment, i.e., locomotion motivated by exploration [79]. Both general locomotor activity and anxiety-related behaviors were assessed as the mice were placed individually inside an open field arena (45 × 45 × 30 cm) and were allowed to move freely for 10 min [80]. Mice were introduced into the center area of the arena (23 × 23 cm) and given 5 min habituation before actual behaviors recording. The overall distance moved inside the complete arena and time spent in the center and periphery was recorded for 10 min using CCD camera-assisted motion tracking equipment and software program (EthoVision 3.1, Noldus Information Technology, The Netherlands). Measurements obtained included total distance traveled and center and periphery time spent for 5 min. After each testing sessions, chambers we cleaned with 70% ethanol, and enough time was allowed for ethanol evaporation and odor dissipation. When evaluating the results, more time spent in the center indicated low levels of anxiety-like behaviors and total distance travelled represented locomotor activity [77,81,82].

### 4.5. Sample Preparation and LC-MS/MS Conditions

Perfusion was done on anesthetized animals using 1X PBS as described previously [44,76]. Different regions of the brain were snap frozen. Frozen tissues were then weighed and homogenized in a 10-fold volume of acetonitrile. For 50mg of tissue, 500 µL of acetonitrile and Internal standard mix were added and homogenized. Homogenates were centrifuged at 15,000 rcf for 15 min at 4 °C. Tissue levels of dopamine, acetylcholine, histamine, and their metabolites were assessed using LC-MS/MS. LC-MS/MS analysis was conducted utilizing Waters BEH C18 COLUMN (2.1 × 100 mm, 1.7 µm) using Waters Acquity UPLC Binary Solvent Manager and FTN Sample Manager. The system was run in gradient mode with mobile phase A consisting of LC-MS GRADE WATER + 0.1% FORMIC ACID and mobile phase B consisting of 100% ACETONITRILE. Mobile phase was duly filtered through 0.2 µm filter and degassed ultrasonically for 15 min prior to use. Separations were performed at room temperature with 0.3 mL/min flow rate gradient starting with 90% of A for up to 0.5 min. Then, % of A was decreased gradually until it reached 20% at 2.5 min after which at 3 min %A was brought back to the initial condition and left for equilibration for 2 more minutes before the next injection (total 5 min run time). The injection volume was kept at 1 µL. Mass spectrometric detection was performed in multiple reaction monitoring (MRM) mode with positive electrospray ionization (ESI+) on Xevo TQS mass spectrometer (Waters, Milford, MA) equipped with an electrospray ionization (ESI) source and triple quadrupole mass analyzer. MRM transitions and instrument parameters were set by tuning the instrument by infusing each analyte. MRM transitions taken for quantification were 146.2 > 87.1 for Acetylcholine, 112.2 > 95.2 for Histamine, 154.1 > 137.2 for Dopamine, 150.2 > 91.3 for Acetylcholine D4, and 16.3 > 99.3 for Histamine D4. Capillary Voltage, Source Offset, Source Temperature, De solvation Temperature, Cone Gas Flow, De solvation Gas Flow, Collision Gas Flow, Nebulizer Gas Flow were set as 3.0 (kV), 60.0 (V), 150 (°C), 550 (°C), 150 (L/Hr), 1000 (L/Hr), 0.13 (mL/min), and 7 (Bar), correspondingly. Analyte corresponding cone voltage (V) and collision voltage (eV) were set as 25 and 16 for Acetylcholine, 25 and 13 for Histamine, 16 and 10 for dopamine, 25 and 16 for Acetylcholine D4(IS), and 25 and 14 for Histamine d4(IS), respectively.

### 4.6. Statistics

Separate two-way analysis of variance ANOVAs (strain: B6, BTBR treatment: vehicle, 2.5, 5, 10 mg/kg ST-2223) as the between-subjects factor was carried out for behavioral and biochemical assessments. A significant interaction was followed by Tukey post hoc tests to determine significant treatment differences in both strains. For statistical comparisons, the software package SPSS 25.0 (IBM Middle East, Dubai, UAE) was used. *p* values less than 0.05 were considered statistically significant.

## 5. Conclusions

In conclusion, we identified the ability of ST-2223 to enhance histaminergic and modulate dopaminergic activity in BTBR mice. The potential effect of ST-2223 on cognitive deficits related to sociability impairments was confirmed by measuring HA, DA, and ACh in several unique brain regions. The mechanism by which the social behavior is improved following chronic systemic management with ST-2223 may be defined with the functionality of ST-2223 to modulate the brain levels of HA, DA, and ACh in the prefrontal cortex, cerebellum, striatum, and hippocampus through antagonist interaction of ST-2223 with histamine H_3_ auto- and heteroreceptors expressed on histaminergic, dopaminergic, and cholinergic neurons, respectively. The consequences discovered substantial perturbations of neurotransmitters in the brain of BTBR mice, which was modulated by ST-2223 (5 mg) comparable to the ARP, and consequent amelioration of social deficits in treated mice. Given the reality that there are not any FDA-approved powerful treatments for the core symptoms of ASD, subsequent identification of novel remedies such as the multiple-active H3/D2/D3 receptor antagonist ST-2223 is crucial.

## 6. Study Limitations and Future Directions

A limitation of our study is that it did not include female mice based on the idea that females are intrinsically more variable than males due to the estrous cycle, which might influence their behaviors. Nonetheless, this research question is interesting and is worth further research. In addition, reduced sample size was used for ethical reasons; however, a larger sample size is recommended in future studies to avoid affecting the power of the study. Additionally, the effect of test compound ST-2223 at a dose between 2.5 mg/kg and 5 mg/kg was not studied, as such data may suggest a lower effective dose that may be useful in designing more informed clinical trials. However, and collectively, these findings are valuable for and can inform future studies to exacerbate the pharmacological effects of this class of multiple-active drugs in different animal models of ASD with an adequate number of female and male mice. This will provide sufficient evidence with the aim to suggest a possible related therapeutic approach that could improve the quality of ASD interventions.

## Figures and Tables

**Table 1:** In vitro affinities of the multi-active ligand ST-2223 at different histamine and dopamine receptor subtypes and enzymes.

ST-2223	
Target Tested	*K*_i_ (nM)
*h*H_3_R ^a^	4.6
*h*H_1_R ^b^	85.2
*h*D_2_R ^c^	19.8
*h*D_3_R ^c^	2.0
*h*D_1_R ^d^	564
*h*D_5_R ^d^	5064
*Ee*AChE ^e^	1000 (<60% inhibition)
*Eq*BChE ^f^	1000 (<30% inhibition)

^a^ [^3^H]*N*^α^-methylhistamine binding assay, performed with cell membrane preparation of human embryonic kidney (HEK) cells stably expressing the *h*H_3_R (n = 4) [35,36]. ^b^ [^3^H]pyrilamine binding assay, performed with cell membrane preparation of Chinese hamster ovary (CHO) cells stably expressing the *h*H_1_R (n = 2). ^c,d^ Displacement assay was carried out as described previously, using membrane suspension of cell lines stably expressing the human dopamine *h*D_1_Rs and *h*D_5_Rs (HEK) against [^3^H]SCH23390 and *h*D_2_SRs, *h*D_3_Rs (CHO) using [^3^H]spiperone (n = 3) [35,36]. ^e^ AChE: Acetylcholine esterase; *Ee*; electric eel (preliminary data); ^f^ BuChE: Butyrylcholinesterase; *Eq*: equine (preliminary data) (n = 2) [37].

**Table 2:** Summary of the multiple effects provided by ST-2223 on the levels of neurotransmitters quantified by LC-MS/MS in different regions of BTBR mice brain.

		BTBR Mice											
of BTBR vs. B6		Histamine				Dopamine				Acetylcholine			
		VEH	ST-2223 (5 mg)	ST-2223 (5 mg) + RAM	ARP (1 mg/kg)	VEH	ST-2223 (5 mg)	ST-2223 (5 mg)+ RAM	ARP (1 mg)	VEH	ST-2223 (5 mg)	ST-2223 (5 mg)+ RAM	ARP (1 mg)
	Prefrontal cortex	↔	↔	↔	↔	↓ ^a^	↑ ^b^	↑↑ ^c^	↔	↓ ^a^	↑ ^b^	↓ ^c^	↑ ^b^
	Cerebellum	↓ ^a^	↑ ^b^	↓ ^c^	↑ ^b^	↔	↓ ^b^	↑↑ ^c^	↓^b^	↓ ^a^	↔	↔	↑ ^b^
	Striatum	↓ ^a^	↑ ^b^	↓ ^c^	↔	↓ ^a^	↑ ^b^	↑↑ ^c^	↔	↔	↑ ^b^	↓ ^c^	↑ ^b^
	Hippocampus	↓ ^a^	↔	↔	↑ ^b^	↑ ^a^	↔	↑↑ ^c^	↓^b^	↓ ^a^	↑ ^b^	↓ ^c^	↔

^a^ *p* < 0.05 as compared to VEH-treated B6 mice. ^b^ *p* < 0.05 as compared to VEH-treated BTBR mice. ^c^ *p* < 0.05 as compared to ST 2223 (5 mg/kg) treated BTBR mice. VEH: vehicle; **↔**: No significant alterations; ↑: significant increase; ↑↑: additional significant increase vs. ST-2223-treated mice; ↓: significant decrease.

## Data Availability

Data is contained within the article.
